# How rash and eschar came to clinical attention in scrub typhus and Japanese spotted fever

**DOI:** 10.1371/journal.pntd.0014377

**Published:** 2026-05-20

**Authors:** Eiichiro Sando, Ikkoh Yasuda

**Affiliations:** 1 Department of Infection Control, Fukushima Medical University, Fukushima, Japan; 2 Department of General Internal Medicine, Kameda Medical Center, Kamogawa, Japan; Fukuoka University Hospital: Fukuoka Daigaku Byoin, JAPAN

## Abstract

**Background:**

Early diagnosis of scrub typhus and Japanese spotted fever remains challenging because initial manifestations are often nonspecific. In endemic areas, nearly 30% of patients were not correctly diagnosed at the first visit to a participating site. Previous studies have mainly treated rash and eschar as present-or-absent findings. We examined how rash and eschar came to clinical attention in patients correctly diagnosed at the first visit to a participating site and in those diagnosed later.

**Methodology/principal findings:**

We analyzed 217 patients with scrub typhus or Japanese spotted fever from three healthcare facilities in an endemic region of Japan. Because first-visit documentation was incomplete in many delayed-diagnosis cases, recognition-pattern variables were abstracted at the time the correct diagnosis was established. Among these patients, 156 (71.9%) were correctly diagnosed at the first visit to a participating site and 61 (28.1%) were diagnosed after one or more subsequent visits. In multivariable analysis, correct first-visit diagnosis was associated with rash reported as a chief complaint (adjusted odds ratio [aOR] 4.95, 95% confidence interval [CI] 2.07–11.84), rash elicited during history taking (aOR 5.86, 95% CI 2.80–12.27), eschar identified on physical examination (aOR 2.80, 95% CI 1.19–6.58), and eschar elicited during history taking, which was exclusively observed among patients diagnosed at the first visit to a participating site. Age 75 years or older was associated with lower odds of correct first-visit diagnosis (aOR 0.40, 95% CI 0.21–0.75).

**Conclusions/significance:**

In endemic settings, patients correctly diagnosed at the first visit to a participating site more often had rash and eschar brought to clinical attention in specific ways. Directed inquiry about skin symptoms and careful examination for eschar may help recognition in routine care.

## Author summary

Scrub typhus and Japanese spotted fever, mite- and tick-borne bacterial infections, can usually be treated effectively, but they are often hard to recognize early because their first symptoms resemble those of many common illnesses. We studied 217 patients in an endemic region of Japan to examine whether correct diagnosis when patients first came to one of the study hospitals or clinics was related to how rash and eschar (a small skin lesion at the site of a mite or tick bite) came to clinical attention. Patients were more likely to receive the correct diagnosis at that visit when rash was mentioned by the patient, identified through the clinician’s questions, or when an eschar was found during examination. Notably, eschar was identified in many patients whose diagnosis was delayed, suggesting that its presence alone is not sufficient; it must be actively looked for. Older patients were less likely to be diagnosed correctly at the first visit to a participating site, but rash recognition remained associated with correct first-visit diagnosis in this group as well. These findings suggest that directed inquiry about skin symptoms and careful examination for eschar may support earlier recognition in all patients in endemic settings, including older adults.

## Introduction

Scrub typhus (tsutsugamushi disease; ST) and Japanese spotted fever (JSF) are endemic rickettsial diseases in Japan [1,2]. Early recognition is important because timely antimicrobial therapy is associated with better outcomes [3,4]. However, even in endemic areas, nearly 30% of patients were not correctly diagnosed at the first visit to a participating site [5].

The clinical presentation of these infections overlaps with that of many other infectious and inflammatory conditions, which makes early diagnosis difficult [6]. Rash and eschar can provide useful clues, but they may not be reported by patients, may not be elicited during history taking, or may not be identified on examination [5].

Previous clinical studies have generally evaluated rash and eschar as conventional clinical variables, typically recording whether they were present-or-absent, rather than how they came to clinical attention [4,7]. In practice, whether rash or eschar contributes to correct diagnosis may depend on how these findings come to clinical attention, not merely on their presence or absence. We therefore examined how rash and eschar came to clinical attention when the diagnosis was established, and whether these recognition patterns differed between patients diagnosed at the first visit to a participating site and those diagnosed later.

## Methods

### Ethics statement

The study was approved by the Institutional Review Board of Fukushima Medical University (No. 2022–165), as the coordinating institution for this multi-site retrospective analysis. The dataset consisted of existing de-identified clinical information collected during routine care between 2004 and 2015, for which an opt-out procedure had been implemented at each institution. The requirement for additional consent was waived by the review board.

### Study design and setting

We analyzed case series data from the southern Boso Peninsula, a highly endemic area in central Japan for both ST and JSF, from 2004 to 2015 [5]. In the parent study, 31 JSF cases occurred primarily from April to October, whereas 188 ST cases occurred mostly in November and December [5]. The dataset comprised retrospective cases (January 1, 2004–December 31, 2010) and prospectively collected cases (January 1, 2011–December 31, 2015) from three facilities: Kameda Medical Center (865 acute beds), Awa Regional Medical Center (149 beds), and the outpatient-focused Kameda Family Clinic–Tateyama. The same formal case definitions and laboratory diagnostic framework were applied to cases from both periods. No standardized case report form or structured symptom-elicitation protocol for rickettsial diseases was in place during either period.

### Participants and case definition

We included patients diagnosed with ST and JSF who presented to the participating sites. Diagnoses were defined according to the criteria used in the parent study [5], as follows. Cases were classified as confirmed if PCR from the eschar was positive or if a 4-fold or greater rise in IgM or IgG titer was observed in paired sera. Cases were classified as probable if the initial IgM titer was 80 or greater for ST or JSF. Both confirmed and probable cases were included in the analysis. Possible cases, murine typhus, and concurrent JSF/ST infections were excluded, as in the parent study [5]. Severe fever with thrombocytopenia syndrome, another important tick-borne infection in Japan, was also not included because our focus was on rickettsial diseases in which rash and eschar are central diagnostic findings. This infection had not been reported in the study region during the study period [8].

Serological testing was performed by indirect immunofluorescence or immunoperoxidase assay at commercial and public health laboratories. Supplementary testing included locally prevalent *O. tsutsugamushi* serotypes in the Boso Peninsula, such as Irie/Kawasaki and Hirano/Kuroki; Shimokoshi was included in selected immunoperoxidase assays. Detailed laboratory procedures have been described previously [5]. Nested PCR assays targeting the 56-kDa antigen of *Orientia tsutsugamushi* and the 17-kDa genus-common antigen of *Rickettsia japonica* were performed on eschar specimens at the Chiba Prefectural Institute of Public Health or Kameda Medical Center [5].

Of the 219 cases in the parent study, 2 ST cases were excluded because it could not be determined from medical records whether the correct diagnosis was made at the first visit or at a subsequent visit, leaving 217 patients for analysis.

### Outcomes and variables

The primary outcome was correct diagnosis at the first visit to a participating site versus delayed diagnosis after that visit. Patients correctly diagnosed with ST or JSF at the first visit to a participating site formed the “diagnosed-at-first-visit group.” Those not correctly diagnosed at the first visit to a participating site but correctly diagnosed after one or more subsequent visits formed the “delayed-diagnosis group.” Because first-visit documentation was incomplete and the initial visit sometimes occurred outside the participating sites, predictor variables were abstracted at the time the correct diagnosis was established. Accordingly, the recognition patterns analyzed in this study reflect how rash and eschar came to clinical attention when the correct diagnosis was established, rather than necessarily what was present, documented, or detectable at the initial visit. Variables included demographic factors, prior visit status, clinical department, illness duration, and laboratory values. Direct visit was defined as first presentation to a participating site without any prior evaluation at another clinic or hospital. Non-direct visit was defined as prior evaluation at another clinic or hospital before presentation to a participating site, regardless of whether a referral letter was provided. Recognition patterns for rash and eschar were each categorized as follows: reported as a chief complaint, elicited during history taking, or identified on physical examination.

### Statistical analysis

Categorical variables were compared using Fisher’s exact test and continuous variables using the Mann–Whitney U test [9,10]. We used logistic regression to estimate adjusted odds ratios (aORs) with 95% confidence intervals (CIs) for associations between recognition patterns and diagnosis at the first visit to a participating site versus delayed diagnosis [11]. Because variables were assessed at the time the correct diagnosis was established rather than at the first visit, these analyses were exploratory. Adjustment variables included age as a continuous variable, sex, and clinical department at the time of correct diagnosis. When age ≥ 75 years was evaluated as an exposure variable, continuous age was omitted from the adjustment set. When clinical department was evaluated as an exposure variable, clinical department was omitted from the adjustment set. Missing data were handled by complete case analysis for each variable. We also performed exploratory supplementary analyses stratified by study period, age group (<75 years versus ≥75 years), and direct visit status. To provide a more detailed descriptive assessment of clinical department, we grouped departments into general internal medicine, frontline/generalist medicine, dermatology, and other specialties. Two-sided *p* < 0.05 was considered statistically significant. All analyses were performed using Stata version 18.0 (StataCorp, College Station, Texas).

## Results

Among 217 patients with rickettsial diseases, 156 (71.9%) were correctly diagnosed at the first visit to a participating site and 61 (28.1%) were diagnosed after one or more subsequent visits.

### Baseline characteristics

Table 1 summarizes participant characteristics at the time the correct diagnosis was established. Compared with the diagnosed-at-first-visit group, the delayed-diagnosis group more often included patients aged 75 years or older, direct-visit patients, and patients with a longer interval from symptom onset to correct diagnosis. Among 217 patients, 140 were in the direct-visit group and 77 were in the non-direct-visit group. Correct first-visit diagnosis was more common in the non-direct-visit group than in the direct-visit group (63/77 [81.8%] vs 93/140 [66.4%], p = 0.018).

**Table 1:** Baseline characteristics of the diagnosed-at-first-visit group and the delayed-diagnosis group.

	Total (N = 217)	Diagnosed-at-first-visit group (n = 156)	Delayed-diagnosis group (n = 61)	*p* value
Rickettsial diseases				
Scrub typhus, n (%)	186 (85.7%)	132 (84.6%)	54 (88.5%)	0.52
Japanese spotted fever, n (%)	31 (14.3%)	24 (15.4%)	7 (11.5%)	0.52
Age				
Median age, years (IQR)	68 (58–76)	67 (58–76)	72 (58–80)	0.12
Age group (≥75 years), n (%)	69 (31.8%)	41 (26.3%)	28 (45.9%)	0.009
Sex female, n (%)	101 (46.5%)	71 (45.5%)	30 (49.2%)	0.65
General internal medicine, n (%)	132 (60.8%)	101 (64.7%)	31 (50.8%)	0.065
Direct visit, n (%)	140 (64.5%)	93 (59.6%)	47 (77.0%)	0.018
Median time from symptom onset to correct diagnosis, days (IQR)	6 (3–7)	5 (3–7)	6 (5–9)	<0.001

Diagnosed-at-first-visit group: patients correctly diagnosed at their first visit to a participating site; Delayed-diagnosis group: patients not correctly diagnosed at the first visit to a participating site but correctly diagnosed after one or more subsequent visits; Direct visit: first presentation to a participating site without prior evaluation at another clinic or hospital. Median time from symptom onset to correct diagnosis: time from symptom onset to the date on which the correct diagnosis of ST or JSF was established; IQR: interquartile range. Group comparisons used Fisher’s exact test (categorical) and the Mann–Whitney U test (continuous). All variables were assessed at the time the correct diagnosis was established.

### First documented incorrect diagnoses

Table 2 shows the first documented incorrect diagnoses before the correct diagnosis was established among 54 of the 61 patients in the delayed-diagnosis group. These incorrect diagnoses were heterogeneous: upper respiratory viral infection (n = 15, 27.8%), bacterial infections such as sepsis, urinary tract infection, or pneumonia (n = 12, 22.2%), and fever of unknown origin (n = 11, 20.4%) were most common. Among these 54 patients, the clinical department associated with the first documented incorrect diagnosis could be determined in 25 patients. These cases most often involved frontline care settings, including emergency medicine, general internal medicine, and the emergency department internal medicine duty service. Of the 54 patients, 40 were in the direct-visit group and 14 were in the non-direct-visit group. Because the number of non-direct-visit patients was small, we did not perform a formal statistical comparison of diagnostic categories between the two groups. Descriptively, upper respiratory viral infection, bacterial infections, and fever of unknown origin were the predominant categories in both groups.

**Table 2:** First documented incorrect diagnoses in the delayed-diagnosis group.

Incorrect diagnosis category	n (%) (N = 54)
Viral infections	
URI, pharyngitis	15 (27.8%)
Influenza	1 (1.9%)
Infectious mononucleosis	1 (1.9%)
Viral infection or drug reaction	1 (1.9%)
Bacterial infections	
Sepsis, UTI, or pneumonia	12 (22.2%)
Infective endocarditis	2 (3.7%)
Group A streptococcal pharyngitis	1 (1.9%)
Drug-related diagnoses	
Drug fever, drug rash	2 (3.7%)
Fever of unknown origin	11 (20.4%)
Other diagnoses	
Enteritis	1 (1.9%)
Headache	1 (1.9%)
Vertigo	1 (1.9%)
Stroke	1 (1.9%)
Malignant rheumatoid arthritis	1 (1.9%)
SJS	1 (1.9%)
Cholecystitis	1 (1.9%)
Neuroleptic malignant syndrome	1 (1.9%)

First documented incorrect diagnoses could be classified in 54 of 61 patients. Categories were assigned based on the first documented incorrect diagnosis available before the correct diagnosis was established, including diagnoses documented at participating sites or in referral information from other medical facilities, supplemented by patient-reported diagnoses when documented in the medical record. URI: upper respiratory infection; UTI: urinary tract infection; SJS: Stevens-Johnson syndrome.

### Recognition patterns and correct first-visit diagnosis

**Fig 1:**
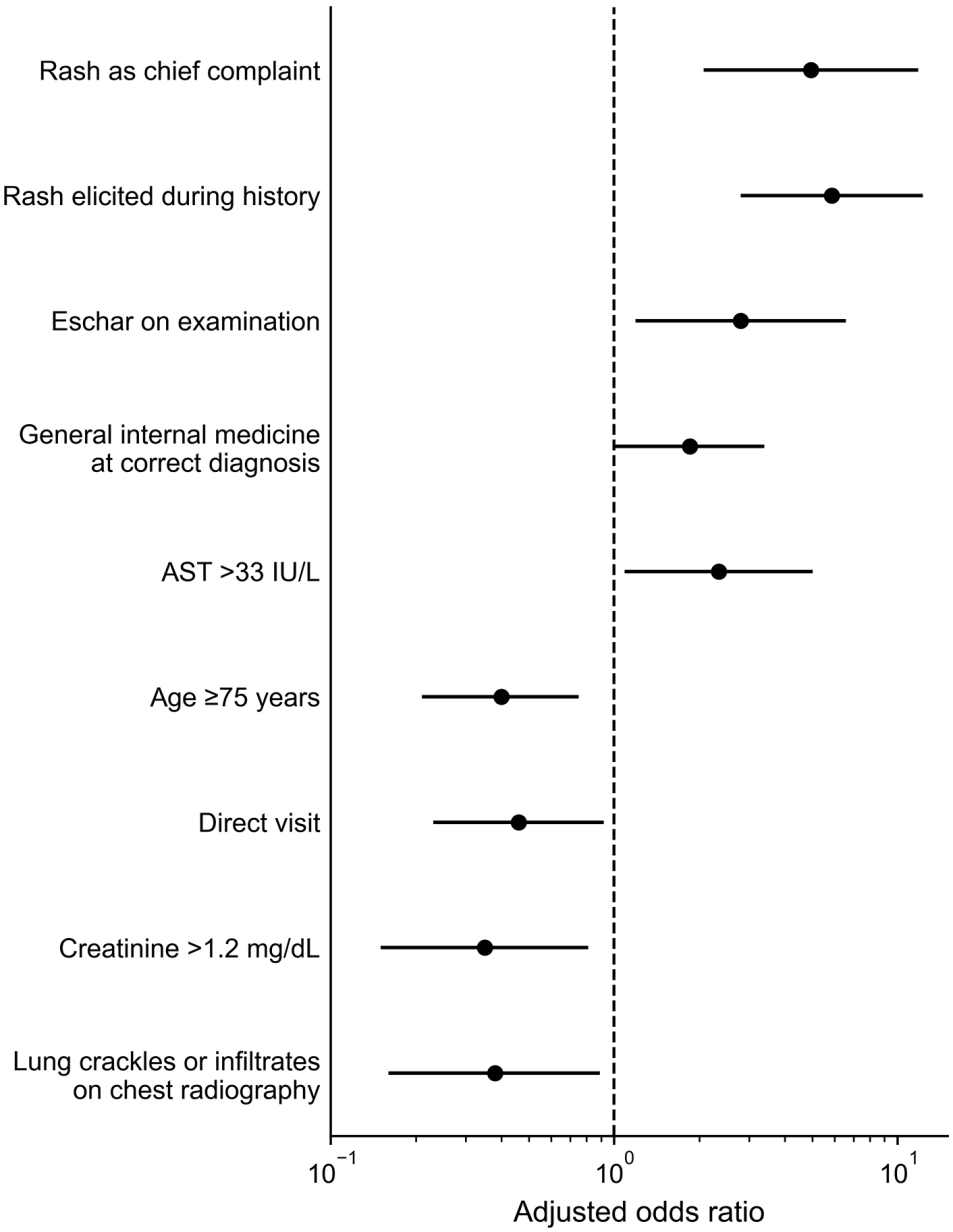
Variables associated with correct diagnosis at the first visit to a participating site. Forest plot of adjusted odds ratios (aORs) with 95% confidence intervals (CIs) for variables associated with correct first-visit diagnosis among 217 patients with rickettsial diseases. The vertical dashed line indicates an aOR of 1.0. Rash as the chief complaint, rash elicited during history taking, eschar detected on physical examination, evaluation by general internal medicine at the time of correct diagnosis, and AST > 33 IU/L were associated with correct first-visit diagnosis, whereas age ≥ 75 years, direct visit, creatinine >1.2 mg/dL, and lung crackles or infiltrates on chest radiography were associated with lower odds of correct first-visit diagnosis. aORs were estimated using logistic regression models adjusted for age as a continuous variable, sex, and clinical department at the time of correct diagnosis. When age ≥ 75 years was evaluated as an exposure variable, continuous age was omitted from the adjustment set; when clinical department was evaluated as an exposure variable, clinical department was omitted from the adjustment set. Because variables were assessed at the time the correct diagnosis was established, these associations should be interpreted cautiously. Full results are shown in S1 Table.

In multivariable analysis, several recognition patterns for rash and eschar differed between the diagnosed-at-first-visit group and the delayed-diagnosis group (Fig 1). Among the skin-related variables, rash reported as a chief complaint (aOR 4.95, 95% CI 2.07–11.84), rash elicited during history taking (aOR 5.86, 95% CI 2.80–12.27), and eschar identified on physical examination (aOR 2.80, 95% CI 1.19–6.58) were each independently associated with correct first-visit diagnosis. Eschar was identified on physical examination in 91.5% of the diagnosed-at-first-visit group and in 78.0% of the delayed-diagnosis group. Age 75 years or older was associated with lower odds of correct first-visit diagnosis (aOR 0.40, 95% CI 0.21–0.75). Several additional factors were associated with higher odds of correct first-visit diagnosis: evaluation by general internal medicine at the time of correct diagnosis (aOR 1.85, 95% CI 1.01–3.39), and AST > 33 IU/L (aOR 2.34, 95% CI 1.09–5.02). Conversely, direct visit (aOR 0.46, 95% CI 0.23–0.92), creatinine >1.2 mg/dL (aOR 0.35, 95% CI 0.15–0.81), and lung crackles or infiltrates on chest radiography (aOR 0.38, 95% CI 0.16–0.89) were associated with lower odds of correct first-visit diagnosis. Full adjusted results are shown in S1 Table.

All patients in whom an eschar was elicited during history taking were correctly diagnosed at the first visit to a participating site. Therefore, an adjusted odds ratio could not be estimated for this variable. In additional exploratory analyses, rash-related recognition patterns remained positively associated with correct first-visit diagnosis across age groups and study periods, although several subgroup estimates were less precise because of smaller sample sizes (S2 and S3 Tables). Baseline characteristics by direct-visit status are shown in S4 Table. Similar analyses stratified by direct-visit status are shown in S5 Table. The proportion of correct first-visit diagnosis also differed across grouped clinical departments (S6 Table).

## Discussion

In this endemic setting, the way rash and eschar came to clinical attention differed between the diagnosed-at-first-visit group and the delayed-diagnosis group. Specifically, compared with the delayed-diagnosis group, the diagnosed-at-first-visit group more often had rash recognized as a chief complaint or during history taking, and eschar recognized on physical examination. Notably, eschar was identified on physical examination in 78.0% of patients in the delayed-diagnosis group at the time the correct diagnosis was established. This pattern is consistent with the parent study, in which patients more often noticed rash than eschar spontaneously [5]. Together, these observations suggest that early recognition may depend not only on the presence of rash or eschar, but also on whether these findings are actively sought during clinical evaluation. These supplementary analyses also suggested that the overall direction of the rash-related associations was broadly preserved across study periods, age groups, and direct-visit groups, although several subgroup estimates were imprecise.

The broad spectrum of incorrect diagnoses documented before the correct diagnosis was established reflects the diagnostic challenge that precedes the recognition of rash and eschar. These diagnoses were heterogeneous, consistent with the nonspecific early presentation of rickettsial diseases (Table 2) [4,12]. Fever was common in both groups but was not independently associated with correct first-visit diagnosis, highlighting its limited discriminatory value in this setting. Older age was associated with lower odds of correct first-visit diagnosis, though the underlying reasons remain unclear. Possible explanations include less specific symptom reporting in older adults, which may reflect pre-existing cognitive impairment, subtle changes in attention or consciousness during acute illness, as well as greater diagnostic competition from common infections in this age group and under-recognition of skin findings during initial frontline evaluations. These interpretations remain speculative.

In endemic areas, targeted questioning about rash and thorough skin inspection for eschar are practical steps toward earlier recognition of ST and JSF in routine care. In ST, eschars may occur in soft skin folds and covered areas such as the axillae, inguinal region, genital area, and trunk [13]. In JSF, attention to the extremities and to rash involving the palms and soles may aid recognition [5].

Lung crackles or infiltrates on chest radiography and elevated creatinine were associated with lower odds of correct first-visit diagnosis. Because these variables were assessed at the time the correct diagnosis was established rather than at the initial visit, we could not determine whether they contributed to initial misdiagnosis, reflected subsequent disease progression, or both. Rickettsial diseases can involve the lungs and kidneys [14–16]. From a clinical perspective, however, such findings may have anchored clinicians toward alternative diagnoses such as pneumonia, urinary tract infection, or sepsis. These findings do not exclude ST or JSF from consideration in endemic settings. In contrast, elevated AST was associated with higher odds of correct first-visit diagnosis. AST was assessed at the time the correct diagnosis was established and may reflect disease severity or diagnostic context rather than an independent contributor to early recognition.

In Japan, general internal medicine is a hospital-based specialty that evaluates undifferentiated and complex medical conditions, including fever of unknown origin. In this setting, it typically receives referrals from primary care physicians and emergency departments. The associations with direct visit and general internal medicine likely reflect the diagnostic pathway rather than direct causal effects. Some non-direct-visit patients, and some patients seen by general internal medicine, may have arrived with accumulated clinical information from prior evaluation. For example, failure to respond to initial antibiotics or a persistent fever with rash may have narrowed the differential diagnosis toward rickettsial disease. Consistent with this interpretation, correct first-visit diagnosis at the participating sites was more common in the non-direct-visit group than in the direct-visit group (81.8% vs 66.4%, p = 0.018). However, some non-direct-visit patients who were classified as being in the diagnosed-at-first-visit group had already experienced a delay in diagnosis from their first point of care. These variables should therefore be interpreted as indicators of diagnostic context, not specialty-specific advantages.

We analyzed ST and JSF together because our aim was to evaluate shared processes related to first-visit recognition, not to distinguish disease-specific clinical features. Both infections also require similar initial clinical consideration in endemic settings.

This study has several limitations. Most importantly, predictor variables were assessed at the time of correct diagnosis, not at the first visit. We therefore could not determine whether these findings had been present but overlooked or became more apparent over time. In the delayed-diagnosis group, the initial visit sometimes occurred outside the participating sites, and even when first-visit records were available, documentation of clinical findings was often incomplete when rickettsial disease was not initially suspected. Second, because this secondary analysis relied on medical-record review across retrospective and prospectively collected cases, the availability and consistency of clinical documentation were limited. For the same reason, data on empirical antimicrobial treatment at the initial visit were not available in a sufficiently complete and standardized form across all patients in the delayed-diagnosis group. Third, direct visit and clinical department likely reflected diagnostic context rather than independent causal effects, particularly because non-direct-visit patients may have arrived at participating sites with additional clinical information or a narrowed differential diagnosis. Fourth, because this study was conducted in endemic settings in Japan, the findings may not be fully generalizable to non-endemic settings or to health systems with different patterns of health care access. Fifth, ST and JSF were analyzed together, and disease-specific differences may not have been fully captured. Additionally, the relatively small number of patients in the delayed-diagnosis group (n = 61) may have limited statistical power, particularly for subgroup analyses. Although the dataset included both retrospective and prospective periods, no uniformly applied protocol for eliciting rash, eschar, or related symptoms was in place during either period. Differences in data capture intensity between the two periods therefore cannot be entirely excluded. For non-direct-visit patients, information about the specialty of the physician at the first point of care outside the participating sites was not consistently available, precluding a systematic analysis of this factor. In addition, the content of referral documentation from prior physicians was not systematically captured in the study dataset, and the potential contribution of information shared from outside physicians to the initial diagnostic impression at the participating sites could not be assessed. Future prospective studies that systematically document rash and eschar at the initial visit, regardless of clinical suspicion, would be needed to determine whether recognition of these findings at the first encounter directly improves diagnostic accuracy. Despite these limitations, this study suggests that the diagnostic process may depend not only on the presence of rash and eschar but also on how these findings come to clinical attention.

## Conclusions

In endemic settings, patients with ST or JSF who were correctly diagnosed at the first visit to a participating site more often had rash and eschar brought to clinical attention in specific ways. Directed inquiry about skin symptoms and careful examination for eschar remain clinically important in routine care.

## Supporting information

S1 Table Adjusted Odds Ratios (aORs) for Variables Associated with Correct First-Visit Diagnosis. (DOCX)

S2 Table Stratified Analyses of Variables Associated with Correct First-Visit Diagnosis in Patients Younger than 75 Years and Those Aged 75 Years or Older. (DOCX)

S3 Table Stratified Analyses of Variables Associated with Correct First-Visit Diagnosis in the Retrospective and Prospective Study Periods. (DOCX)

S4 Table Baseline Characteristics of Patients by Direct-Visit Status. (DOCX)

S5 Table Stratified Analyses of Variables Associated with Correct First-Visit Diagnosis According to Direct-Visit Status. (DOCX)

S6 Table Proportions of Correct First-Visit Diagnosis According to Grouped Clinical Departments. (DOCX)
