# Supplementary material for: How rash and eschar came to clinical attention in scrub typhus and Japanese spotted fever
Source: PLoS Negl Trop Dis. 2026 May 20;20(5):e0014377. doi: 10.1371/journal.pntd.0014377 (PMC13197070; doi:10.1371/journal.pntd.0014377)
Supplement: S1 Table — (DOCX) [file pntd.0014377.s001.docx]

**S1 Table. Adjusted Odds Ratios (aORs) for Variables Associated with Correct First-Visit Diagnosis.**

|  |  |  | Diagnosed-at-first-visit group,  n (%) | Delayed-diagnosis group,  n (%) | N | aOR (95% CI) |
| --- | --- | --- | --- | --- | --- | --- |
| Demographic and clinical context | | |  |  |  |  |
|  | Age ≥75 years | | 41 (26.3%) | 28 (45.9%) | 217 | 0.40 (0.21–0.75) |
|  | Female sex | | 71 (45.5%) | 30 (49.2%) | 217 | 0.86 (0.47–1.57) |
|  | Department | |  |  |  |  |
|  |  | General internal medicine | 101 (64.7%) | 31 (50.8%) | 217 | 1.85 (1.01–3.39) |
|  |  | Direct visit | 93 (59.6%) | 47 (77.0%) | 217 | 0.46 (0.23–0.92) |
|  |  | Time from symptom onset to correct diagnosis <5 days | 74 (48.1%) | 14 (23.7%) | 213 | 3.23 (1.61–6.49) |
| Risk factors | | |  |  |  |  |
|  | High exposure | |  |  |  |  |
|  |  | No exposure | 11 (8.5%) | 8 (15.1%) | 182 | Reference |
|  |  | Residence in mountainous area, no forest entry | 54 (41.9%) | 15 (28.3%) | 182 | 2.34 (0.77–7.05) |
|  |  | Entry into bamboo groves | 11 (8.5%) | 2 (3.8%) | 182 | 3.79 (0.64–22.56) |
|  |  | Fieldwork (gardening, rice fields, bushes, etc.) | 53 (41.1%) | 28 (52.8%) | 182 | 1.38 (0.50–3.84) |
| Chief complaint | | |  |  |  |  |
|  |  | Fever | 116 (74.4%) | 43 (70.5%) | 217 | 1.16 (0.59–2.28) |
|  |  | Rash | 59 (37.8%) | 7 (11.5%) | 217 | 4.95 (2.07–11.84) |
|  |  | Eschar | 4 (2.6%) | 1 (1.6%) | 217 | 1.86 (0.19–17.94) |
|  |  | Headache | 19 (12.2%) | 11 (18.0%) | 217 | 0.52 (0.23–1.22) |
|  |  | Fatigue | 26 (16.7%) | 12 (19.7%) | 217 | 0.79 (0.36–1.70) |
| History taking | | |  |  |  |  |
|  |  | Fever | 125 (83.9%) | 48 (78.7%) | 210 | 1.30 (0.60–2.83) |
|  |  | Rash | 80 (58.0%) | 12 (20.0%) | 198 | 5.86 (2.80–12.27) |
|  |  | Eschar | 21 (15.4%) | 0 (0.0%) | 194 | Not estimable |
|  |  | Headache | 55 (49.5%) | 24 (61.5%) | 150 | 0.54 (0.24–1.22) |
|  |  | Fatigue | 83 (83.0%) | 29 (90.6%) | 132 | 0.53 (0.14–1.97) |
| Physical examination | | |  |  |  |  |
|  |  | Body temperature >37.5°C | 110 (73.3%) | 43 (71.7%) | 210 | 1.03 (0.52–2.04) |
|  |  | Hypotension (SBP <90 mmHg or vasopressor use) | 14 (9.0%) | 6 (9.8%) | 217 | 0.88 (0.31–2.46) |
|  |  | Heart rate >120 beats/min | 10 (7.6%) | 4 (6.9%) | 189 | 1.51 (0.43–5.30) |
|  |  | Respiratory rate >20 breaths/min | 39 (42.9%) | 12 (35.3%) | 125 | 1.40 (0.60–3.29) |
|  |  | Altered mental status | 12 (7.7%) | 7 (11.5%) | 217 | 0.57 (0.21–1.58) |
|  |  | Rash | 152 (98.1%) | 57 (93.4%) | 216 | 2.98 (0.62–14.25) |
|  |  | Rash-localized | 1 (0.6%) | 2 (3.4%) | 215 | 0.17 (0.01–1.92) |
|  |  | Rash-purpura | 12 (7.9%) | 3 (5.0%) | 211 | 1.75 (0.47–6.54) |
|  |  | Rash-palms/soles | 28 (18.7%) | 6 (10.0%) | 210 | 1.94 (0.74–5.08) |
|  |  | Eschar | 140 (91.5%) | 46 (78.0%) | 212 | 2.80 (1.19–6.58) |
|  |  | Lymphadenopathy | 46 (34.3%) | 14 (28.0%) | 184 | 1.36 (0.63–2.94) |
| Laboratory and imaging tests | | |  |  |  |  |
|  |  | WBC >9800/μL | 19 (12.3%) | 9 (14.8%) | 216 | 0.73 (0.31–1.76) |
|  |  | Hb <11 g/dL (female), <13.5 g/dL (male) | 20 (13.0%) | 13 (21.3%) | 215 | 0.49 (0.21–1.13) |
|  |  | Platelet <130,000/μL | 59 (38.1%) | 22 (36.1%) | 216 | 1.09 (0.58–2.06) |
|  |  | Albumin <3.4 g/dL | 38 (33.9%) | 12 (32.4%) | 149 | 1.27 (0.55–2.95) |
|  |  | AST >33 IU/L | 136 (87.7%) | 45 (75.0%) | 215 | 2.34 (1.09–5.02) |
|  |  | ALT >42 IU/L | 87 (56.1%) | 28 (46.7%) | 215 | 1.35 (0.73–2.51) |
|  |  | LDH >229 IU/L | 147 (96.1%) | 60 (98.4%) | 214 | 0.43 (0.05–3.70) |
|  |  | Total bilirubin >1.0 mg/dL | 14 (9.4%) | 8 (13.8%) | 207 | 0.57 (0.22–1.49) |
|  |  | Direct bilirubin >0.4 mg/dL | 7 (6.7%) | 4 (8.5%) | 152 | 0.66 (0.18–2.48) |
|  |  | Creatine Kinase >150 IU/L | 42 (31.6%) | 21 (40.4%) | 185 | 0.62 (0.31–1.24) |
|  |  | BUN >22 mg/dL | 32 (20.6%) | 17 (28.8%) | 214 | 0.60 (0.29–1.25) |
|  |  | Creatinine >1.2 mg/dL | 18 (11.6%) | 14 (23.7%) | 214 | 0.35 (0.15–0.81) |
|  |  | Sodium <135 mEq/L | 69 (45.1%) | 26 (44.1%) | 212 | 1.00 (0.53–1.89) |
|  |  | Chloride <98 mEq/L | 39 (27.5%) | 15 (25.9%) | 200 | 0.98 (0.48–2.02) |
|  |  | C-reactive protein >10 mg/dL | 36 (23.5%) | 12 (20.3%) | 212 | 1.30 (0.61–2.78) |
|  |  | Urine protein (Qualitative) | 98 (76.0%) | 43 (78.2%) | 184 | 0.60 (0.25–1.42) |
|  |  | Urine blood (Qualitative) | 103 (79.8%) | 47 (85.5%) | 184 | 0.88 (0.35–2.19) |
|  |  | Lung crackles or infiltrates on chest radiography | 15 (9.6%) | 14 (23.0%) | 217 | 0.38 (0.16–0.89) |
| Severity | | |  |  |  |  |
|  |  | quick SOFA score ≥2 | 16 (10.3%) | 8 (13.1%) | 217 | 0.67 (0.26–1.73) |
|  |  | Hospitalization | 90 (57.7%) | 40 (65.6%) | 217 | 0.60 (0.30–1.19) |

A logistic regression model was used to estimate adjusted odds ratios (aORs) with 95% confidence intervals (CIs) for factors associated with correct diagnosis at the first visit to a participating site. Models were adjusted for age as a continuous variable, sex, and clinical department at the time of correct diagnosis. When age ≥75 years was evaluated as an exposure variable, continuous age was omitted from the adjustment set; when clinical department was evaluated as an exposure variable, clinical department was omitted from the adjustment set. The "Fieldwork" category within the high exposure group included one imported case from South Korea. The anatomical distribution of localized rash was not systematically recorded in the dataset. Cases with an eschar elicited during history taking were all diagnosed at the first visit; therefore, the adjusted odds ratio was not estimable. Delayed-diagnosis group: patients not correctly diagnosed at the first visit to a participating site but correctly diagnosed after one or more subsequent visits; N: number of cases with valid data for each variable, out of a total of 217 cases; General internal medicine: evaluation by general internal medicine at the time of correct diagnosis; Direct visit: first presentation to a participating site without prior evaluation at another clinic or hospital; Time from symptom onset to correct diagnosis: time from symptom onset to the date on which the correct diagnosis of ST or JSF was established; SBP: systolic blood pressure; WBC: white blood cell count; Hb: hemoglobin; AST: aspartate aminotransferase; ALT: alanine aminotransferase; LDH: lactate dehydrogenase; BUN: blood urea nitrogen.
