# Supplementary material for: How rash and eschar came to clinical attention in scrub typhus and Japanese spotted fever
Source: PLoS Negl Trop Dis. 2026 May 20;20(5):e0014377. doi: 10.1371/journal.pntd.0014377 (PMC13197070; doi:10.1371/journal.pntd.0014377)
Supplement: S2 Table — (DOCX) [file pntd.0014377.s002.docx]

**S2 Table. Stratified Analyses of Variables Associated with Correct First-Visit Diagnosis in Patients Younger than 75 Years and Those Aged 75 Years or Older.**

**Panel A. Patients Younger than 75 Years**

|  |  |  | Diagnosed-at-first-visit group,  n (%) | Delayed-diagnosis group,  n (%) | N | aOR (95% CI) |
| --- | --- | --- | --- | --- | --- | --- |
| Clinical context | | |  |  |  |  |
|  |  | General internal medicine | 72 (62.6%) | 16 (48.5%) | 148 | 1.77 (0.81–3.87) |
|  |  | Direct visit | 66 (57.4%) | 26 (78.8%) | 148 | 0.37 (0.15–0.93) |
| Recognition patterns | | |  |  |  |  |
|  |  | Rash as a chief complaint | 47 (40.9%) | 6 (18.2%) | 148 | 3.52 (1.31–9.48) |
|  |  | Rash elicited during history taking | 61 (58.7%) | 9 (28.1%) | 136 | 4.03 (1.66–9.82) |
|  |  | Eschar on physical examination | 102 (91.1%) | 25 (80.6%) | 143 | 2.20 (0.71–6.88) |
| Laboratory and imaging tests | | |  |  |  |  |
|  |  | AST >33 IU/L | 98 (86.0%) | 24 (75.0%) | 146 | 2.00 (0.76–5.24) |
|  |  | Creatinine >1.2 mg/dL | 9 (7.9%) | 6 (19.4%) | 145 | 0.28 (0.09–0.92) |
|  |  | Lung crackles or infiltrates on chest radiography | 8 (7.0%) | 2 (6.1%) | 148 | 1.22 (0.24–6.23) |

**Panel B. Patients Aged 75 Years or Older**

|  |  |  | Diagnosed-at-first-visit group,  n (%) | Delayed-diagnosis group,  n (%) | N | aOR (95% CI) |
| --- | --- | --- | --- | --- | --- | --- |
| Clinical context | | |  |  |  |  |
|  |  | General internal medicine | 29 (70.7%) | 15 (53.6%) | 69 | 2.11 (0.77–5.79) |
|  |  | Direct visit | 27 (65.9%) | 21 (75.0%) | 69 | 0.66 (0.21–2.01) |
| Recognition patterns | | |  |  |  |  |
|  |  | Rash as a chief complaint | 12 (29.3%) | 1 (3.6%) | 69 | 10.48 (1.26–87.37) |
|  |  | Rash elicited during history taking | 19 (55.9%) | 3 (10.7%) | 62 | 13.31 (3.01–58.84) |
|  |  | Eschar on physical examination | 38 (92.7%) | 21 (75.0%) | 69 | 4.25 (0.97–18.54) |
| Laboratory and imaging tests | | |  |  |  |  |
|  |  | AST >33 IU/L | 38 (92.7%) | 21 (75.0%) | 69 | 4.36 (0.97–19.62) |
|  |  | Creatinine >1.2 mg/dL | 9 (22.0%) | 8 (28.6%) | 69 | 0.56 (0.17–1.83) |
|  |  | Lung crackles or infiltrates on chest radiography | 7 (17.1%) | 12 (42.9%) | 69 | 0.28 (0.09–0.86) |

A logistic regression model was used to estimate adjusted odds ratios (aORs) with 95% confidence intervals (CIs) for factors associated with correct first-visit diagnosis within each age stratum. Models were adjusted for sex and clinical department at the time of correct diagnosis; for the clinical department variable itself, models were adjusted for sex only. Diagnosed-at-first-visit group: patients correctly diagnosed at their first visit to a participating site; Delayed-diagnosis group: patients not correctly diagnosed at the first visit to a participating site but correctly diagnosed after one or more subsequent visits; N: number of cases with valid data for each variable within each age stratum; Direct visit: first presentation to a participating site without prior evaluation at another clinic or hospital.
