# Supplementary material for: How rash and eschar came to clinical attention in scrub typhus and Japanese spotted fever
Source: PLoS Negl Trop Dis. 2026 May 20;20(5):e0014377. doi: 10.1371/journal.pntd.0014377 (PMC13197070; doi:10.1371/journal.pntd.0014377)
Supplement: S3 Table — (DOCX) [file pntd.0014377.s003.docx]

**S3 Table. Stratified Analyses of Variables Associated with Correct First-Visit Diagnosis in the Retrospective and Prospective Study Periods.**

**Panel A. Retrospective Period (2004**–**2010)**

|  |  |  | Diagnosed-at-first-visit group,  n (%) | Delayed-diagnosis group,  n (%) | N | aOR (95% CI) |
| --- | --- | --- | --- | --- | --- | --- |
| Clinical context | | |  |  |  |  |
|  |  | General internal medicine | 53 (53.0%) | 19 (43.2%) | 144 | 1.51 (0.74–3.10) |
|  |  | Direct visit | 58 (58.0%) | 32 (72.7%) | 144 | 0.54 (0.25–1.18) |
| Recognition patterns | | |  |  |  |  |
|  |  | Rash as a chief complaint | 38 (38.0%) | 6 (13.6%) | 144 | 4.01 (1.53–10.54) |
|  |  | Rash elicited during history taking | 57 (58.2%) | 11 (25.0%) | 142 | 4.42 (1.97–9.92) |
|  |  | Eschar on physical examination | 92 (92.9%) | 31 (73.8%) | 141 | 4.56 (1.61–12.94) |
| Laboratory and imaging tests | | |  |  |  |  |
|  |  | AST >33 IU/L | 86 (86.9%) | 32 (72.7%) | 143 | 2.46 (1.01–5.98) |
|  |  | Creatinine >1.2 mg/dL | 7 (7.1%) | 7 (16.3%) | 142 | 0.34 (0.11–1.10) |
|  |  | Lung crackles or infiltrates on chest radiography | 12 (12.0%) | 10 (22.7%) | 144 | 0.45 (0.17–1.21) |

**Panel B. Prospective Period (2011**–**2015)**

|  |  |  | Diagnosed-at-first-visit group,  n (%) | Delayed-diagnosis group,  n (%) | N | aOR (95% CI) |
| --- | --- | --- | --- | --- | --- | --- |
| Clinical context | | |  |  |  |  |
|  |  | General internal medicine | 48 (85.7%) | 12 (70.6%) | 73 | 2.77 (0.74–10.40) |
|  |  | Direct visit | 35 (62.5%) | 15 (88.2%) | 73 | 0.26 (0.05–1.28) |
| Recognition patterns | | |  |  |  |  |
|  |  | Rash as a chief complaint | 21 (37.5%) | 1 (5.9%) | 73 | 13.04 (1.34–126.70) |
|  |  | Rash elicited during history taking | 23 (57.5%) | 1 (6.3%) | 56 | 20.73 (2.32–185.32) |
|  |  | Eschar on physical examination | 48 (88.9%) | 15 (88.2%) | 71 | 0.73 (0.12–4.45) |
| Laboratory and imaging tests | | |  |  |  |  |
|  |  | AST >33 IU/L | 50 (89.3%) | 13 (81.3%) | 72 | 1.82 (0.38–8.62) |
|  |  | Creatinine >1.2 mg/dL | 11 (19.6%) | 7 (43.8%) | 72 | 0.30 (0.08–1.14) |
|  |  | Lung crackles or infiltrates on chest radiography | 3 (5.4%) | 4 (23.5%) | 73 | 0.24 (0.04–1.36) |

A logistic regression model was used to estimate adjusted odds ratios (aORs) with 95% confidence intervals (CIs) for factors associated with correct first-visit diagnosis within each study period. Models were adjusted for age as a continuous variable, sex, and clinical department at the time of correct diagnosis; when the clinical department variable itself was evaluated as an exposure variable, clinical department was omitted from the adjustment set. Diagnosed-at-first-visit group: patients correctly diagnosed at their first visit to a participating site; Delayed-diagnosis group: patients not correctly diagnosed at the first visit to a participating site but correctly diagnosed after one or more subsequent visits; N: number of cases with valid data for each variable within each study period; Direct visit: first presentation to a participating site without prior evaluation at another clinic or hospital.
