# Supplementary material for: How rash and eschar came to clinical attention in scrub typhus and Japanese spotted fever
Source: PLoS Negl Trop Dis. 2026 May 20;20(5):e0014377. doi: 10.1371/journal.pntd.0014377 (PMC13197070; doi:10.1371/journal.pntd.0014377)
Supplement: S4 Table — (DOCX) [file pntd.0014377.s004.docx]

**S4 Table. Baseline Characteristics of Patients by Direct-Visit Status.**

| **Variable** | **Non-direct-visit group (n=77)** | **Direct-visit group (n=140)** | **p value** |
| --- | --- | --- | --- |
| Age, median (IQR), years | 66 (57–76) | 68.5 (58–78) | 0.099 |
| Female sex, n (%) | 40 (51.9%) | 61 (43.6%) | 0.257 |
| Age ≥75 years, n (%) | 21 (27.3%) | 48 (34.3%) | 0.361 |
| Scrub typhus, n (%) | 67 (87.0%) | 119 (85.0%) | 0.840 |
| Japanese spotted fever, n (%) | 10 (13.0%) | 21 (15.0%) | 0.840 |
| Correct first-visit diagnosis, n (%) | 63 (81.8%) | 93 (66.4%) | 0.018 |

Non-direct-visit group: patients who had been evaluated at another clinic or hospital before presentation to a participating site. Direct-visit group: patients who first presented to a participating site without prior evaluation at another clinic or hospital. IQR: interquartile range. p values were calculated using the Mann–Whitney U test for age and Fisher’s exact test for categorical variables.
