# Supplementary material for: How rash and eschar came to clinical attention in scrub typhus and Japanese spotted fever
Source: PLoS Negl Trop Dis. 2026 May 20;20(5):e0014377. doi: 10.1371/journal.pntd.0014377 (PMC13197070; doi:10.1371/journal.pntd.0014377)
Supplement: S5 Table — (DOCX) [file pntd.0014377.s005.docx]

**S5 Table. Stratified Analyses of Variables Associated with Correct First-Visit Diagnosis According to Direct-Visit Status.**

**Panel A. Direct-Visit Group**

|  |  |  | Diagnosed-at-first-visit group,  n (%) | Delayed-diagnosis group,  n (%) | N | aOR (95% CI) |
| --- | --- | --- | --- | --- | --- | --- |
| Clinical context | | |  |  |  |  |
|  |  | General internal medicine | 62 (66.7%) | 19 (40.4%) | 140 | 3.13 (1.50–6.56) |
|  |  | Age ≥75 years | 27 (29.0%) | 21 (44.7%) | 140 | 0.46 (0.21–0.99) |
| Recognition patterns | | |  |  |  |  |
|  |  | Rash as a chief complaint | 33 (35.5%) | 6 (12.8%) | 140 | 4.14 (1.51–11.37) |
|  |  | Rash elicited during history taking | 45 (54.2%) | 10 (21.7%) | 129 | 4.51 (1.91–10.67) |
|  |  | Eschar on physical examination | 84 (91.3%) | 37 (80.4%) | 138 | 1.64 (0.54–4.93) |
| Laboratory and imaging tests | | |  |  |  |  |
|  |  | AST >33 IU/L | 75 (81.5%) | 34 (73.9%) | 138 | 1.58 (0.65–3.82) |
|  |  | Creatinine >1.2 mg/dL | 12 (13.0%) | 14 (30.4%) | 137 | 0.27 (0.10–0.70) |
|  |  | Lung crackles or infiltrates on chest radiography | 8 (8.6%) | 11 (23.4%) | 140 | 0.31 (0.10–0.95) |

**Panel B. Non-Direct-Visit Group**

|  |  |  | Diagnosed-at-first-visit group,  n (%) | Delayed-diagnosis group,  n (%) | N | aOR (95% CI) |
| --- | --- | --- | --- | --- | --- | --- |
| Clinical context | | |  |  |  |  |
|  |  | General internal medicine | 39 (61.9%) | 12 (85.7%) | 77 | 0.23 (0.04–1.17) |
|  |  | Age ≥75 years | 14 (22.2%) | 7 (50.0%) | 77 | 0.36 (0.10–1.29) |
| Recognition patterns | | |  |  |  |  |
|  |  | Rash as a chief complaint | 26 (41.3%) | 1 (7.1%) | 77 | 7.35 (0.86–63.02) |
|  |  | Rash elicited during history taking | 35 (63.6%) | 2 (14.3%) | 69 | 7.20 (1.39–37.34) |
|  |  | Eschar on physical examination | 56 (91.8%) | 9 (69.2%) | 74 | 4.04 (0.79–20.66) |
| Laboratory and imaging tests | | |  |  |  |  |
|  |  | AST >33 IU/L | 61 (96.8%) | 11 (78.6%) | 77 | 8.45 (0.93–76.54) |
|  |  | Creatinine >1.2 mg/dL | 6 (9.5%) | 0 (0.0%) | 77 | Not estimable* |
|  |  | Lung crackles or infiltrates on chest radiography | 7 (11.1%) | 3 (21.4%) | 77 | 0.61 (0.12–3.23) |

A logistic regression model was used to estimate adjusted odds ratios (aORs) with 95% confidence intervals (CIs) for factors associated with correct first-visit diagnosis within each direct-visit group. Models were adjusted for age as a continuous variable, sex, and clinical department at the time of correct diagnosis. When age ≥75 years was evaluated as an exposure variable, continuous age was omitted from the adjustment set; when clinical department was evaluated as an exposure variable, clinical department was omitted from the adjustment set. Diagnosed-at-first-visit group: patients correctly diagnosed at their first visit to a participating site; Delayed-diagnosis group: patients not correctly diagnosed at the first visit to a participating site but correctly diagnosed after one or more subsequent visits; N: number of cases with valid data for each variable within each direct-visit group; Direct visit: first presentation to a participating site without prior evaluation at another clinic or hospital. *Not estimable because creatinine >1.2 mg/dL perfectly predicted correct first-visit diagnosis in the non-direct-visit group.
