# Supplementary material for: How rash and eschar came to clinical attention in scrub typhus and Japanese spotted fever
Source: PLoS Negl Trop Dis. 2026 May 20;20(5):e0014377. doi: 10.1371/journal.pntd.0014377 (PMC13197070; doi:10.1371/journal.pntd.0014377)
Supplement: S6 Table — (DOCX) [file pntd.0014377.s006.docx]

**S6 Table. Proportions of Correct First-Visit Diagnosis According to Grouped Clinical Departments.**

| **Grouped clinical department** | Diagnosed-at-first-visit group,  n (%) | Delayed-diagnosis group,  n (%) | Total |
| --- | --- | --- | --- |
| Dermatology | 19 (82.6%) | 4 (17.4%) | 23 |
| Frontline/generalist | 30 (66.7%) | 15 (33.3%) | 45 |
| General internal medicine | 101 (76.5%) | 31 (23.5%) | 132 |
| Other specialties | 6 (35.3%) | 11 (64.7%) | 17 |
| Total | 156 (71.9%) | 61 (28.1%) | 217 |

Fisher’s exact p = 0.004

Clinical departments were grouped according to the department at the time the correct diagnosis was established: general internal medicine; frontline/generalist medicine included internal medicine other than general internal medicine, family medicine, emergency medicine, and infectious diseases; dermatology; and other specialties. Diagnosed-at-first-visit group: patients correctly diagnosed at their first visit to a participating site. Delayed-diagnosis group: patients not correctly diagnosed at the first visit to a participating site but correctly diagnosed after one or more subsequent visits. Percentages are row percentages.
